# Entry into Nursing: An Ethnographic Study of Newly Qualified Nurses Taking on the Nursing Role in a Hospital Setting

**DOI:** 10.1155/2012/690348

**Published:** 2012-09-24

**Authors:** Mari Skancke Bjerknes, Ida Torunn Bjørk

**Affiliations:** ^1^Faculty of Nursing, Oslo and Akershus University College, Pilestredet 52, N-0167 Oslo, Norway; ^2^Department of Nursing Science, University of Oslo, Postboks 1153, Blindern, 0318 Oslo, Norway

## Abstract

The transition from student to working nurse has long been recognized as challenging. This paper presents the findings of research into the opportunities and limitations encountered by newly qualified nurses when taking on the nursing role. The study had an ethnographic design. Observation, interviews, and document analysis were used to gain insight into nurses' daily work from the perspective of recently graduated nurses. Thirteen nurses were monitored closely during their first year in a hospital setting in Norway. These new nurses generally entered the field with empathy for their patients, enthusiasm for the profession, and readiness to learn more about being a good nurse. However, their more experienced colleagues seemed to neither respect nor nurture this attitude. The new nurses experienced heavier responsibilities than expected, fragmentation of patient care, and stressful interactions with colleagues. The lack of a supportive work environment and role models increased the new nurses' experience of overwhelming responsibility in their daily work situations. The nurses learned to cope the hard way, despite the organizational culture, not because of it. Adjusting the profession's expectations of new nurses, and offering good role models and more comprehensive support programmes, would markedly ease the transition for new nurses.

## 1. Introduction

The transition from student to working nurse has long been recognized as difficult and has attracted the attention of researchers for decades [1–5]. Various studies have shown that new graduates are inadequately prepared for their role as nurses, and they are ineffectually oriented to the work place [6–8]. The nursing discipline and nurse education are currently experiencing the compound pressure of expectations, which may lead to deteriorating conditions for nursing practice. The clinical field, under increasing pressure to be profitable, efficient, and effective, is demanding education programmes that produce graduate nurses who are work ready from day one [9]. Professional discussions of the deteriorating conditions for nursing as a caring profession have also cited the apparent movement among nurses away from both a wish for human contact and a willingness to comfort the sick and suffering for its own sake [10, 11]. Such studies indicate that the conditions for nursing remain challenging in terms of increased pressure in the work environment and new nurses' readiness and willingness to care for patients. This situation inspired us to investigate empirically how new nurses are taking on the nursing role in hospitals.

## 2. Background

Until the 1980s, nurse education in many Western countries was based predominantly on an apprenticeship model, in which student nurses were salaried members of the nursing work force in hospitals [12–14]. The three-year programme in schools of nursing had no formal academic recognition. In the early 1990s, major reforms in many countries redefined both the process and the product of nurse education. The aim was to equip student nurses with the necessary knowledge and skills to assume the role of graduates, and to achieve academic recognition [6, 15, 16]. Efforts to improve the quality of study programmes and subsequent nursing practice led to a shift towards research-based teaching. However, this drift towards academia seems to have gone too far, resulting in complaints about weak practical skills and the lack of caring abilities among recently trained nurses [17, 18].

New nurses' qualifications cannot be viewed solely in terms of education policy and reform. Changes in the organization and delivery of health care have a great impact on nurse education. The current nursing culture in hospital wards is characterized by a stronger treatment culture and greater demands for efficiency than in previous years [19]. In all Western countries, several reforms have led to higher stress levels in hospital wards, more specialized medical treatments and technologies, and shorter patient stays [6, 7, 20]. A related factor in Norway is that the health-care system has undergone substantial organizational changes in the past 10–15 years [21].

It is evident from the literature that new nurses do not experience a supportive practice environment and regularly encounter unrealistic expectations [8, 22]. Boswell and Wilhoit [23] found that nurses may need as long as one year after graduation to feel competent. Many health-care organizations have introduced mentorship strategies as a way of increasing work readiness, minimizing the effects of reality shock, and reducing graduate nurse attrition [8, 22]. Kramer [1] used the term “reality shock” in nursing to describe how school-bred values conflict with work-world values, a concept based on the foundational tenets of culture shock. Reports of this transition in nursing have proliferated in recent years [5, 7, 16, 19, 24]. The term “transition” refers to the processes of learning and adjustment that a new staff member undergoes to acquire the skills, knowledge, and values required to become an effective member of the health-care team [7, 25]. This process involves shifting from one set of expected positional behaviours to another in a specific social system: it incorporates a socialization process or “rites of passage,” in which the graduates absorb and adopt the language, culture, and rules of the work place. In a Canadian study, Duchscher [16] emphasized that it was the quality of the transitional experience that was likely to influence graduate nurses' self-confidence and retention in nursing. That study provided insight into the socialization process of nurses by asking them to reflect on their first six months in the profession. A clear conflict emerged between the nurses' desire to deliver good nursing and their lack of competence, time, and energy to do so.

The expectations encountered by new graduates in Norway today are usually the same as those from before the 1990s, despite the changes in nurse education and in the content and context of hospital nursing. Graduates are offered some form of orientation programme, usually lasting two or three weeks. Although the content varies, these programmes predominantly focus on patients' diagnoses, hospital routines, and medical treatments characteristic of the ward [26]. Graduates in hospitals are supposed to have a mentor among the clinical nursing staff, who provides some support during the first four or five months in the ward. However, because of the hectic clinical setting, new nurses spend much of their time on their own [27].

Research conducted into this transition using qualitative methodologies has investigated the nature of the transition process and the values of new nurses [16, 24], but little attention has been given to the reality of new nurses' clinical practice. Missing from the literature is an insider's perspective on new nurses' experience when they take on the nursing role in a hospital setting. Knowledge of new graduates' experiences in their daily work situations should help improve understanding of the challenges they face in their role as nurses [28]. In this paper, we present the findings of a study that focused on the question: *What opportunities and limitations do newly qualified nurses encounter when taking on the nursing role?*

## 3. Methods

### 3.1. Design

An ethnographic design was used, drawing upon observations, interviews, and the analysis of documents. This was a qualitative design, monitoring the nurses in their encounters with colleagues, physicians, and patients in clinical situations. The combination of different data sources is often highly productive, and the resulting analysis and findings can be more complete when a number of different information sources are used to explore a question [29].

### 3.2. Sample and Setting

The setting was one gynaecological and one orthopaedic ward of equal size (30 beds) in a Norwegian university hospital, during one year of fieldwork. A meeting was held to give the participants and their colleagues information about the study. Written information was also distributed to all new nurses in the wards. For nurses to be included, they had to have been qualified for about three months when the study started. Although the sample selected was a convenience sample, it was also purposeful, because the participants were ideal in terms of having some experience of being a new nurse. The sample comprised 13 new graduates; all were women, in their early to mid-twenties (mean age 25 years), and from Norwegian backgrounds.

### 3.3. Data Collection

The nurses were monitored through observation and interviews in the context of the wards. Reflection notes written by the new graduates about their work and caring experiences were included as data. The researcher (MSB) observed the nurses' activities at meetings, in staff rooms, in patient rooms, in ancillary rooms, in the tea room, and in the corridors at different times during the day and evening. The nurses were followed individually. No single period of observation exceeded four hours. The nurses' shifts provided the starting points and the structure of the fieldwork. During observations, the researcher wore the same uniform as the nurses in order to merge into the setting and make it easier to be accepted as a natural participant in the ward. Observational notes were written out in full immediately after each period of observation so that no valuable information would be lost or forgotten. The recorded interviews were transcribed during the research process.

### 3.4. Trustworthiness of the Data

We followed the guidelines of Lincoln and Guba [30] for establishing the trustworthiness of data. To establish the credibility of the data, the informants were encouraged to share their perspectives on different situations with the researcher. The informants were also asked to read through some of the transcripts, and they were encouraged to comment on their accuracy, correct them as required, and provide supplementary explanations if they felt it was appropriate. Although we acknowledge the criticisms concerning participant checking [29], we felt that the participants themselves should establish the validity of the interpretations. The dependability of the analysis required sufficient arguments and documentation in the text so that the reader could follow the analysis. Finally, the study fulfils the criterion of confirmability when its findings can be applied to contexts beyond the study situation [30].

### 3.5. Data Analysis

The analysis proceeded in parallel with the data collection, as the researchers read through the transcripts repeatedly. Initially, the task was to find concepts that would make sense of the data. According to Benner [31], the nurses' world can never be delineated completely, because although it is historical, contextual, and multifaceted, it can only be grasped in finite, situated terms. We followed the principle of analysing abundant data on a few informants and have provided “thick descriptions” [32]. The main analysis was performed after the end of the year of fieldwork. For this purpose, we relied on a set of analytical techniques [33]. The process started with quite open questions, for example, What do new graduates do? What are they talking about? With whom are they cooperating? We wished to create new ways of looking at the nurses' work in order to interpret everyday situations with a higher degree of abstraction. In this process, more specific questions developed, consistent with themes such as the nurse's responsibility or “being busy.” Other themes increased in importance as they recurred in the nurses' actions and statements. The different data sources were compared for one nurse and across the 13 nurses. We searched for similarities and contrasts in the ways in which the individual and the group as a whole experienced their relationships with colleagues and patients in different situations.

### 3.6. Ethical Considerations

The study was approved by the hospital research ethics committee. Informed consent was obtained from each participant before data collection commenced. The participants were assured that any data about their participation would be treated confidentially. Because the researcher was an external person, with no employment links to the hospital, the participants could feel free to be honest in dialogues about their work and their work environment. The participants were informed that they were free to withdraw from the study at any time without any explanation. According to common practice, the nurses always asked the patients' permission to bring a researcher into their rooms.

## 4. Findings

In this section, the two central findings are presented from the perspectives of Tina and Kari. Their experiences represent typical challenges that the nurses reported throughout the entire body of material. The remaining findings give voice to all the nurses in the study.

### 4.1. Taking Care of Patients

“The patients in the ward have many complex diseases and they need regular observation day and night,” Tina told me as I followed her down the corridor. In one room, a young woman lay with her eyes closed and her face towards us. Tina quietly approached the bedside and bent down, whispering: “Are you awake? Can I get you something to drink?” The patient only grunted in reply and did not open her eyes. Her face was pale and she had a kidney bowl on her chest holding some absorbent tissue. While looking at the intravenous catheter and checking the intravenous injection, Tina kept an eye on the patient's face. She stood there for some minutes. After a period of silence, the patient replied, “My mouth is dry, so perhaps just a swallow.” A glass of fresh water was on the bedside table, and Tina carefully supported the patient and helped her take a small mouthful. The patient swallowed some water and spat out the rest. “Just a small swallow, yes, that's good,” Tina said. After helping the patient into a comfortable position, Tina said that she would be back again soon. On the way out, I noticed a soft light in the single room, a glow from the lamp beside the bed, positioned in such a way that it would not bother the patient. I asked Tina about this patient afterwards, and she told me that the patient had a serious disease, hyperemesis gravidarum, which meant that she was constantly sick with nausea and vomiting. The patient would have to stay in bed for months or possibly for her entire pregnancy, and she was not supposed to have anything to drink or eat. “I really feel sorry for this patient, so young and being so sick day after day,” she said. Later in the afternoon, and in between phone calls and her responsibilities for other patients, Tina slipped in to see the young pregnant woman. “I must not disturb her,” she whispered to me. “I have to follow up and see if she is all right, and whether she has vomited.” She helped the patient and offered her a special moisture stick in a glass of iced water to cleanse her mouth, which would give her a sense of tasting water. Tina's caring and sensitive attitude when taking care of the patient was in marked contrast with her much more determined and brisk manner of walking as soon as she was back in the corridor.

Involvement in patient situations gave Tina an opportunity to be close to a person who had complex care needs. Tina experienced herself and her competence in caring in different ways. She used her knowledge and empathy to support the young pregnant woman “just enough” so that the patient would feel taken care of. The patient received the necessary rest and relief from her nausea and vomiting. Tina was pleased because she felt that she had displayed the clinical skills required for the medical treatment, while at the same time comforting and calming the patient. She experienced being responsible for the care of a patient. She was also aware that being responsible for a number of patients at the same time generated a kind of anxiety within her. She said that this was particularly so when she did not have enough time for all her patients during a shift: “On night duty, I have got more time for the patients. I can be there when the day-time's hectic rhythms are not in force any more. And no one cares if I spend a long time with one patient.”

### 4.2. Role of a Team Leader

Kari said she had started to feel more secure in her position, but that acting as a team leader was still an overwhelming responsibility. On one particular day, she was responsible for seven patients with her team. This included all the hands-on work, such as medications, wound care, and many quite specialized procedures. After the early morning report, she sat down to look through trays of paperwork, talk to secretaries, fetch laboratory results, and check physicians' orders and medications. I could hear Kari and two other nurses in the staff room talking about the “stress of getting an overview”, while they were waiting for the physician's round. Kari looked confused, and I asked if there was something special about today. “No,” she said, but the physician coming to perform the doctor's round would be late. Then, why did she persist with the paperwork for so long? When I asked Kari about this afterwards, she told me that the paperwork concerned the need for getting the essential data about the patients. She added that she was reluctant to begin the patients' morning care if she was going to be interrupted in the middle of these activities, and that it was often difficult to finish an assignment that had been started. “In the middle of giving a bed bath to one patient, I often have to leave the patient to give another patient premedication or join the physician's rounds. I feel this is showing disrespect for the patients.”

Finally, it was Kari's turn to follow the physician. The physician seemed busy, barely greeting Kari before he headed for the first patient's room. Kari almost had to run to catch up with him. At the first patient's bedside, the physician asked Kari about the records and started to explain the details of the disease to the patient. It was obvious that the patient did not understand it all. Kari attempted to add something, but the physician turned away and attended briefly to the other patient in the room, before leaving. Kari followed, but she stopped by the first patient's bed to say, “I will come back, and we can talk more.” By the time she was back in the corridor, the physician was already in the next room; he finished his whole round in five minutes. Kari asked the doctor questions about medications and rehabilitation but found the answers unsatisfactory. After the round, Kari prepared some medications, and then she went to visit two patients, checking their intravenous lines and some dressings. She asked the patients how they were feeling, and she seemed to create good relationships with the patients. Back in the corridor, she told me that she would have no time to follow up the patients this morning as she had planned, apart from talking with the patient who looked worried after receiving the physician's information. At the end of the day, I asked Kari about her working relationships with the physicians. She told me that they were not always like that “some are quite friendly, but for the most part, cooperating with them is frustrating.” Then, she blurted out: “It is all too much running after them—on our time.”

In Kari's case, it was clear that the situation required balance between attending to the patients' needs and acquiring the necessary overview before the physician's round. At the same time, she had learned to recognize and take into account patients' particular illness-related problems, and how to communicate her observations to the physician. The experience of being the team leader gave Kari the opportunity to learn what can only be mastered in practice. Despite the difficulties of her situation, Kari felt satisfied when she had made sure that the patients were satisfied, and when, as team leader, she had “got everything done, written the reports, ordered the blood tests, and signed patients out and arranged for social workers and physiotherapists, and everything else that has to be done.”

### 4.3. The Challenge of Being Responsible

The new nurses were unprepared for the myriad of feelings provoked by this responsibility while trapped in administrative routines; they had not been aware of the extent of the work load and its consequences for the quality of their care. One nurse, who considered herself well acquainted with the ward from her student days, noticed a marked difference between being a student and bearing the responsibility of being a nurse. She said that “the main difference between training and being on the job is in fact the responsibility.” This responsibility included making independent decisions, especially during evening and night shifts. The new nurses had high expectations of their own capabilities and felt that they should be able to handle difficult situations. However, there was a discrepancy between their perceptions of their own skills and knowledge and their actual ability to draw on these to assume the responsibility required. This discrepancy was a source of stress on busy days. One nurse wrote in a narrative that she was very much aware that there would be a lot of responsibility, “but I feel there's even more. I feel that it's more and more like nursing on an acute ward as far as the responsibility is concerned.” Many of the patients in the gynaecological ward were women close to their own age, with serious and sometimes life-threatening diseases. When asked to discuss this, the nurses said that the responsibility was difficult when there were time constraints and “you feel that you do not have the resources you need, or the energy itself and the knowledge to tackle what is expected of you. Then it gets tough, and that happens quite frequently.”

### 4.4. Excitement of Learning More

Despite the conflict between patient care and administrative tasks, the new nurses were enthusiastic about learning to do their job well. For many of them, a positive aspect of working in a surgical ward was the variety in their work: this was one of the main reasons that they had begun their careers in a hospital. One nurse was pleased because she had learned that she “wanted to be an angel, but also that this is where it's all happening”. She added: “I want the best of both worlds, to mean something to the patients, but also to learn more.” Another nurse said “That's perhaps what gives most of the meaning to being a nurse, that's what we work for.” She told me about a time when she was happy because a patient told her in the morning that the painkiller she had given him had helped and that he had slept well for the rest of the night. In such situations, the nurses felt they had done a good job. They seemed to want intimacy—to be there for the patients—but they also wanted excitement. They found it exciting to learn more about technical procedures and to manage new situations with the patients, especially when they could concentrate on what they were doing without interruption.

### 4.5. Lack of Guidance

The general impression derived from the data was that the new nurses experienced little support or had few opportunities to discuss the challenging situations they encountered in their work. Problems that required practical solutions were given precedence. The bustle in the wards meant that conversations often took place in passing, for example, on the way to the morning care routine. The new graduates' relational experiences with the patients received little attention, and the patients' situations were only rarely the subject of discussion in the care environment.

Resolving the conflict inherent in scheduling and organizing their attention to patients was a challenge for the new nurses, and the fragmentation of their work left them feeling that their more experienced colleagues neither understood nor respected their ideas about nursing. The level of support for the new graduates in this respect varied; for some, it was negligible. Any organized supervision that was available was ear marked for the more senior nurses. One nurse said “If there's anyone who needs guidance, it's us, the newcomers. If only we could talk more with the experienced nurses, then there would be some guidance at least.” Although the quality of mentorship varied, it was perceived as useful when it “included opportunities to work together, formal meetings and completion of paperwork.” The most readily available support was given by the other nurses in the team; such support often involved a mix of informal input, such as discussing a shift before going home, and more structured support, such as help filling out a critical-incident statement. The new nurses seemed to have developed a defensive attitude in response to this “busyness” and lack of support: “You just get on with your job as part of the system. There's not much else we can do.” Although each graduate nurse was supposed to have a mentor for the first three or four months, the system did not work well because their shifts were not coordinated.

## 5. Discussion

The empirical findings of this study both conflict and concur with other relevant research findings. Our study showed that the new nurses were keenly aware of their patients' needs and wanted to give them the best care possible, and that they were concerned about creating good relationships with their patients. This patient-centred caring was also observed in Duchscher's [16] study, in which it was associated with increased knowledge. However, when Rognstad et al. [11] studied students' educational and occupational motivations, they found a high degree of self-centredness. The students were described as typical representatives of the “what's in it for me” generation, whose main concern was to fulfil their own wishes and needs. Tveit [34] moderated this impression in her study. She found that nursing students had an ambivalent attitude: they were motivated towards nursing because they wanted to help the sick, but at the same time, helping others increased their self-understanding and accentuated their own importance. In this respect, Tveit encourages us to perceive self-centredness among students and new nurses as a potential strength. As in the studies of both Tveit [34] and Maben et al. [24], the new nurses in our study wanted to make a difference to their patients.

The nurses' positive energy and interest in improving their skills did not seem to be fully exploited, or valued by their more experienced colleagues. Maben et al. [24] found that their participants' attempts to put their ideals into practice were often hindered by processes designated “organizational sabotage” and “professional sabotage.” Professional sabotage included a lack of support and poor nursing role models. Organizational sabotage included time pressures and excessive work loads. Our findings confirm the presence of this bureaucratic aspect in the work place. The new nurses were sometimes anxious about whether they could meet the profession's expectations, especially when they were responsible for a large number of patients, and particularly on days when they were team leaders. Whereas existing studies have described anxiety (fear of making mistakes) and a desire for ongoing feedback among new nurses [16, 35, 36], the nurses in our study did not seem to worry constantly about making mistakes. They felt a lack of energy and knowledge and wanted support, but they seemed to manage the challenge of assuming responsibility. As team leaders, the nurses experienced different styles of physician cooperation, and felt that they were in an insecure position because the physicians seemed to expect the nurses to be ready for them at any time. This finding confirms those of Duchscher [16], Dyess and Sherman [8], and Martin and Wilson [5], in that the new nurses in their studies frequently experienced less than ideal communication with physicians. The lack of professional confidence that new nurses feel in challenging situations can be heightened when another professional is brusque or expresses displeasure [8]. However, as Leever et al. [37] pointed out, the collaboration between nurses and physicians in a hospital is crucial to the care of individual patients.

Although the nurses in our study did not directly confirm the findings of Kramer [1] in terms of “massive transition,” there was a perceptible “psychological shift,” which seemed to mark the adjustment from being a student to becoming a staff nurse. This adjustment was mainly associated with the recognition that “the main difference between training and being on the job was the responsibility,” in that the new nurses had become answerable for all their decisions in practice, a factor also identified by Maben and Clark [38] and Dearmun [39]. The new graduates wanted to manage, despite the difficulties, and they, therefore, accepted work loads and responsibilities that could provoke feelings of stress. In an effort to conform to the implicit value of being seen to be doing a good job and to “play the organizational game,” the nurses became aware of the importance of “appearing to be busy” [40], which they often manifested in their manner of moving briskly through the corridors. According to Lindberg-Sand [41], this aspect of “reality shock” can be understood as a profound transition experience. She suggested that new nurses' experiences of responsibility are interwoven with the aspect of time, so that the feeling of being responsible increased in tandem with the speed with which the nurses fulfilled their duties. In this context, the new graduates in our study seemed to have adapted to the implicit requirement to be efficient in order to avoid trouble and to earn the respect of their leader and other staff members. Unacknowledged value conflicts and role ambiguities may lie behind the unrealistic expectations held by senior nurses. Ellerton and Gregor [35] and Maben et al. [24] described an incongruence between the personal values of new graduates and the established values in clinical practice settings and questioned the effect on nurses of not having the energy or capacity to realize their ideal of nursing. These conflicting expectations emerged clearly in our study and illustrate the pressures felt by new nurses. According to Duchscher and Cowin [4], the primary challenge for new nurses lies in their struggle to construct a new professional sense of self-confidence that fuses the ideals of their education with the realities of their practice context.

The new nurses in our study were attracted to a job with possibilities for excitement, and for them, working in a surgical ward was about learning more. They saw the job as giving them an opportunity to take care of patients and to learn how to be a good nurse. However, it became apparent that there was no support tailored to their individual learning needs, leading to a dissonance between the support and supervision that they felt they needed. As recent graduates, they had expected that the more experienced nurses would provide guidance as they developed their clinical skills, assist them with decision making, and help them meet their patients' needs and the organizational requirements of the hospital. These findings are consistent with previous studies [5, 24, 36]. Wenger [42] argues for an interaction between the nurses' participation in practical work and a dialogue about this work. It is through practice that students and new graduates can work and form opinions about that work. Our findings support Olson's conclusion [36] that collegiality is highly valued by new nurses today, and that mutually supportive and empathetic relationships with colleagues play an important role in how well new nurses adapt to the culture of nursing [5].

### 5.1. Methodological Considerations

This study explored the perceptions and experience of the reality of practice among 13 new graduate nurses in a Norwegian hospital. Although small, the sample provided an opportunity for observation, in-depth interviews, repeat interviews, and ongoing journal reflections, all of which enriched the study. The questions addressed to the informants were constructed during the fieldwork, and they should be recognized as coming from within the author's personal and professional paradigm of thought. This subjectivity indirectly instilled a conceptual bias in the fieldwork process, although rigorous attempts were made to ensure that the informants directed the type of data collected. The context of the two surgical wards differed, but this issue was not addressed in this study. However, incorporating these contextual differences would not have increased the validity of the data.

All data were collected by the main author. At the time of the study, the author had been a practising acute-care nurse for five years and had previous involvement as a nurse educator. As in all qualitative research, the author's practice experience necessarily influenced both the process and the content of the study. This is a strength of the study, because it offers an insider's perspective on the views of new graduates.

## 6. Conclusions

The research presented in this paper shows that newly graduated nurses are likely to enter nursing practice with empathy for their patients and enthusiasm for their profession. However, this potential in new nurses and their readiness to learn seem to be hindered by organizational and professional limitations, including time pressures and a lack of support. For a transition to be optimal, it must take place in a well-structured ward, with a supportive environment characterized by initiatives that will meet the needs of new nurses during their first year of practice. Such initiatives should include support that extends beyond clinical orientation and basic unit-specific mentoring programmes. The work experiences of new nurses provide substantial information about improvement opportunities at the ward level. These factors have an important impact on new nurses' work practices and on future nurse retention. Further research in this area is required to explore the experience of being constrained within the professional nursing role, the kinds of feelings this issue engenders in newly graduated nurses, and the effects on their future careers.
